# RETRACTION: Efficient One‐Step Induction of Human Umbilical Cord‐Derived Mesenchymal Stem Cells (UC‐MSCs) Produces MSC‐Derived Neurospheres (MSC‐NS) with Unique Transcriptional Profile and Enhanced Neurogenic and Angiogenic Secretomes

**DOI:** 10.1155/sci/9841479

**Published:** 2026-06-12

**Authors:** Stem Cells International

RETRACTION: C. Peng, Y. Li, L. Lu, J. Zhu, H. Li, and J. Hu, “Efficient One‐Step Induction of Human Umbilical Cord‐Derived Mesenchymal Stem Cells (UC‐MSCs) Produces MSC‐Derived Neurospheres (MSC‐NS) with Unique Transcriptional Profile and Enhanced Neurogenic and Angiogenic Secretomes,” *Stem Cells International*, vol. 2019 (2019). https://doi.org/10.1155/2019/9208173.

The above article, published online on 17 December 2019 in Wiley Online Library (wileyonlinelibrary.com), has been retracted following agreement between the Journal’s Associate Editor Tao‐Sheng Li; and John Wiley & Sons Ltd.

The retraction has been agreed following an investigation by the publisher, which highlighted concerns related to Figure 2b in the manuscript. More specifically, the panel “bFGF only” exhibited overlapping features with Figure 1c of another paper by the same author group [1], where it depicts a different cell type.

The authors provided the raw data intended to support the conclusions of the article. Upon the assessment of the provided data, further concerns were identified. As a result of this investigation, the results of the article are no longer considered reliable. Therefore, the article is retracted.

The authors disagree with this retraction.
